# Correction: miR-199a-3p knockdown inhibits dedifferentiated liposarcoma (DDLPS) cell viability and enhances apoptosis through targeting casein kinase-1 alpha (CK1α)

**DOI:** 10.1039/c9ra90070e

**Published:** 2019-10-15

**Authors:** Ye Cao, Jiajia Zheng, Chentao Lv

**Affiliations:** Department of General Surgery, Shanghai Public Health Clinical Center No. 921 Rd. Tongxin Hongkou Shanghai 200083 China y9919002tang@163.com +86-13651613217; Department of General Surgery, Zhongshan Hospital, Fudan University Xuhui Shanghai 200030 China

## Abstract

Correction for ‘miR-199a-3p knockdown inhibits dedifferentiated liposarcoma (DDLPS) cell viability and enhances apoptosis through targeting casein kinase-1 alpha (CK1α)’ by Ye Cao *et al.*, *RSC Adv.*, 2019, **9**, 22755–22763.

The authors regret that the address for affiliation *b* was incorrect in the original article. The correct address is as presented here.

The Royal Society of Chemistry apologises for these errors and any consequent inconvenience to authors and readers.

## Supplementary Material

